# *E2F1* copy number variations in germline and breast cancer: a retrospective study of 222 Italian women

**DOI:** 10.1186/s10020-021-00287-2

**Published:** 2021-03-10

**Authors:** Maria Santa Rocca, Clara Benna, Elena Goldin, Andrea Di Nisio, Luca De Toni, Ilaria Cosci, Alberto Marchet, Donato Nitti, Carlo Foresta

**Affiliations:** 1 Department of Medicine, Unit of Andrology and Reproductive Medicine, University Hospital Padua, Via N. Giustiniani 2, 35128 Padua, Italy; 2 Department of Surgery Oncology and Gastroenterology, University of Padua, Padua, Italy; 3 First Surgical Clinic, University Hospital Padua, Padua, Italy; 4 Breast Unit, Azienda ULSS n.8 Berica, Vicenza, Italy; 5 Veneto Institute of Oncology IOV-IRCCS, Padua, Italy; 6 Multidisciplinary Day-Surgery Unit, University Hospital Padua, Padua, Italy

**Keywords:** Copy number variations, CNV, *E2F1*, Breast cancer, Biomarker

## Abstract

**Background:**

Breast cancer is the most common neoplasia among women in developed countries. The risk factors of breast cancer can be distinguished in modifiable and unmodifiable factors and, among the latter, genetic factors play a key role. Copy number variations (CNVs) are genetic variants that are classified as rare when present in less than 1% of the healthy population. Since rare CNVs are often cause of diseases, over the last years, their contribution in carcinogenesis has become a relevant matter of study. E2F1 is a transcriptional factor that plays an important role in regulating cell cycle and apoptosis. Its double and conflicting role is the reason why it acts both as oncogene and as tumour suppressor, depending on cell context. Since anomalies in expression or in number of copies of *E2F1* have been related to several cancers, we aimed to study number of germline copies of *E2F1* in women with breast cancer in order to better elucidate their contribution as predisposing factor to this tumour.

**Methods:**

We performed, hence, a retrospective study on 222 Italian women with breast cancer recruited from October 2002 to December 2007. TaqMan CNV assay and Real-Time PCR were carried out to analyse, respectively, *E2F1* CNV and *E2F1* expression in the subjects of the study. Chi square test or Fisher’s exact test and Student's t‐test were used to calculate the frequency of CNVs and differences in continuous variables between groups, respectively.

**Results:**

Intriguingly, we found that 10/222 (4.5%) women with breast cancer had more copies than controls (0/200, 0%), furthermore, the number of copies positively correlated with *E2F1* gene expression in breast cancer tissue, suggesting that the constitutive gain of the gene could translate into an increased risk of genomic instability. Additionally, we found that altered *E2F1* copies were present prevalently in the patients with contralateral breast cancer (20%) and all of them had a positive family history, both typically associated with hereditary cancer.

**Conclusions:**

Our findings suggest that copy number variations of *E2F1* might be a susceptibility factor for breast cancer, however, further studies on large cohorts are to be performed in order to better delineate the phenotype linked to the gain of *E2F1* copies.

## Background

Breast cancer (BC) is the most diagnosed neoplastic disease and represents the second cause of cancer-related death, after lung cancer, in women (DeSantis et al. 2019). It is a complex and heterogeneous neoplasia whose aetiology involves several risk factors of genetic, environmental and behavioural origin (Orlandella et al. 2020). Risk factors of BC are generally distinguished in two groups: extrinsic and intrinsic factors. Extrinsic risk factors include obesity, physical activity, alcohol consumption and ionizing radiation exposure, while intrinsic risk factors are unmodifiable parameters such as race, ethnicity, sex, age, early menarche, late menopause, late age at first birth, nulliparity, hormonal factor, family history and genetic mutations (Kaminska et al. 2015). The latter include rare high-risk mutations (*BRCA1* and *BRCA2* genes), more moderate susceptibility variants (*CHEK2* and *ATM* genes) and several still unidentified common susceptibility variants associated with low to moderate increased risk. These known risk factors, however, fail to fully elucidate the high incidence of BC.

Based on the status of estrogen receptor (ER), progesterone receptor (PR) and human epidermal growth factor receptor (HER2), different intrinsic subtypes of BC have been classified as: luminal A, luminal B, HER2-enriched and triple negative breast cancer (TNBC).

ER signalling plays a key role in BC development, in fact, up to 75% of all breast cancers are ERα positive (Allred et al. 2004; Osborne and Shiff 2011; Dai et al. 2016); therefore, the deregulation of downstream target proteins of ER could partially explain the underlying mechanisms of initiation and development of most of BC.

Transcriptional factor E2F1, member of E2F family including both transcriptional activators and repressors, is a downstream target of ER pathway resulting overexpressed in breast cancer tissue (Li et al. 2018).

E2F1 is a transcriptional activator promoting proliferation, following mitogenic stimulation, or apoptosis, as a response to DNA damage. Therefore, E2F1 can act both as an oncogene and as a tumour suppressor, depending upon the cellular context (Engelmann and Pützer 2012). Abnormalities in *E2F1* gene expression or *E2F1* gene amplification have been reported in many types of human cancer (Nelson et al. 2006; Ma et al. 2013; Liang et al. 2016; Kent al. 2017).

Beside gene mutations and hormone receptor (HR) status, copy number variations (CNVs), covering about 12% of whole human genome (Schaschl et al. 2009), have been largely investigated in BC, because of their role as risk factors for several diseases, including tumours (Petrij-Bosch et al.^1997^; Montagna et al. 2003; Casilli et al. 2006; Lesueur et al. 2008; Cybulski et al. 2006; Cybulski et al. 2007; Shlien and Malkin 2009; Kumaran et al. 2017).

In our previous studies, we found that the frequency of germinal CNV of *E2F1* gene in patients with testicular cancer and melanoma was higher compared to healthy controls and, furthermore, the increased number of copies of *E2F1* correlated with an increased gene expression, especially under stress conditions, suggesting germline *E2F1* CNVs as risk factor of these two tumours (Rocca et al., 2017; Rocca et al. 2018).

Based on these recent evidence, the aim of this study was to investigate the frequency of CNV of *E2F1* in 222 Italian women with breast cancer in order to better elucidate the contribution of this structural variant as a potential predisposing factor to breast carcinogenesis.

## Materials and methods

This study was approved by the Ethics Committee of Padova University Hospital (identifier: prot#448).

A total of 222 women of Italian origin were retrospectively selected among patients referred to the First Surgical Clinic, University Hospital of Padua—Veneto Institute of Oncology, Italy for breast cancer. We extracted the clinic-pathological data of treated patients between October 2002 and December 2007, using a prospectively maintained database linked to the biobank of the First Surgical Clinic—University Hospital of Padua, Italy. To be included in the study, each case had to meet the following requirements: (1) histologically confirmed diagnosis of breast cancer or metastasis from breast cancer; (2) pathology-based information on TNM stage; (3) follow-up data (minimum follow up: 6 months); (4) availability of DNA for genotyping purposes (Table 1). Tissue specimens were available for 35 out of 222 patients.

**Table 1:** Description of phenotypic characteristics of patients

Patient characteristics	Mean ± SD No
Age at diagnosis (years)	60 ± 14.5
Menopausal status (N = 222)	
Premenopausal	40
Perimenopausal	27
Menopausal	155
Breast cancer (N = 222)	
Subtype	
HR+	195
Triple negative	6
HER-2+	21
Lymph node status (N = 219)	
Positive	89
Negative	130
Tumour type (N = 222)	
Invasive ductal carcinoma (IDC)	161
Ductal carcinoma in situ (DCIS)	12
Lobular	35
D & L	2
Other	12
Histological grade (N = 199)	
I	21
II	105
III	73
Location (N = 211)	
Unilateral	204
Bilateral	7
Familial history (N = 31)	
Yes	14
No	17
Tumour size (N = 181)	
< 2 cm	107
> 2 cm	74
Vascular invasion (N = 195)	
Yes	35
No	160

200 women, referred to Unit of Andrology and Reproduction Medicine of University Hospital of Padua, with no history of any malignancy were used as controls. All subjects provided written informed consent.

### Copy number variation analysis

Genomic DNA of patients was provided by the above-mentioned biobank. DNA was isolated from peripheral blood leucocytes using QIAamp DNA Blood Mini Kit, according to the manufacturer’s protocol (Qiagen Inc., Hilden, Germany). Copy number variation was evaluated on 20 ng of genomic DNA. Quantitative real-time polymerase chain reaction (PCR) TaqMan Copy Number Assays were performed using three probes targeting different regions of the *E2F1* gene (Hs00576444_cn, Hs01758822_cn and Hs00919582_cn)(Applied Biosystems, Foster City, CA, USA). TaqMan CNV reactions were performed in triplicate using the FAM-dye-labeled assay for *E2F1* and VIC-dye labeled RNase P assay. RNase P assay was used to normalize the genomic DNA input.

An internal DNA resulted with two copies of *E2F1* both by TaqMan Copy Number Assay and array CGH was used as calibrator. Real-time data were collected by the StepOne Plus 2.1 software, and ABI CopyCaller 2.0 software (Thermo Fisher Scientific Inc, Waltham, MA, USA) was used for data analysis. Copy Number ranging from 1.5 to 2.5 were predicted as CNV = 2. Two independent assays were performed for each sample to confirm results.

### RNA isolation, cDNA synthesis and real-time PCR

Total RNA was extracted from 35 breast cancer tissues of patients included in the study using the RNeasy Mini Kit (Qiagen, Hilden, Germany). cDNA was synthesized from 250 ng of total RNA using SuperScript III (Invitrogen, Carlsbad, CA, USA) and random hexamers. Real Time PCR were performed in a 20 µl final volume containing 20 ng of cDNA, 1X Power SYBR Green PCR Master Mix (Applied Biosystem, Foster City, CA, USA), and a mix of forward and reverse primers (1 mmol/l each). The following primers were used: *E2F1*: forward 5′-CATCAGTACCTGGCCGAGAG-3′ and reverse 5′-CCCGGGGATTTCACACCTTT-3′. Human *GAPDH* was used as a housekeeping gene: forward 5′-TCGACAGTCAGCCGCATCTT-3′ and reverse 5′-AGGCGCCCAATACGACCAAA-3′. Real Time PCR was performed on thermocycler StepOne plus (Applied Biosystems, Foster City, CA, USA) and relative quantification was performed using Delta Delta Ct (ΔΔCt) method.

### Statistical analysis

Statistical analysis of the data was conducted with SPSS 21.0 for Windows (SPSS, Chicago, IL). Statistical power was calculated comparing two proportions: the frequency of *E2F1* altered copies in healthy controls and the frequency of *E2F1* altered copies in patients with BC. The α level of significance was set at 0.05.

Differences in the frequency of CNVs between groups were compared using the Chi square test, or Fisher’s exact test when expected frequency was < 5. Differences in continuous variables between groups were analysed by Student's t‐test. P value ≤ 0.05 was considered statistically significant.

## Results

On Table 1 are reported main characteristics of patients. Age did not differ between cases and controls (Age = 60 ± 14.5 and 59 ± 10.4, respectively). Pre-, peri- and post-menopausal women were also comparable between cases and controls (data not shown). In the group of cancer patients, we found a significantly higher portion of women (4.5%, 10/222) with more than two copies of *E2F1*, compared with controls where none of subjects harboured *E2F1* CNVs > 2 (0%, 0/200; *p* = 0.002). The statistical power of this study was 80%.

At this point, we compared patient’s phenotypic characteristics between subjects with CNV > 2 and those with CNV = 2. Table 2 summarises this analysis. In particular, patients with more than 2 copies of *E2F1* had less lymph nodes-positive (*p* = 0.04) and prevalently a bilateral tumour (*p* = 0.04), and, additionally, they had positive BC family history (*p* = 0.001). No difference has emerged for other traits.

**Table 2:** Pathological characteristics in two group of patients. Data are presented as proportion of individuals within each category

	CNV = 2	CNV > 2	*P* value
Age at diagnosis (years)	60.4 ± 14.5	61.4 ± 13.6	0.74
Menopausal status			0.70
Premenopausal	20.9%	10%	
Perimenopausal	3.7%	0.0%	
Menopausal	75.4%	90%	
Estrogen receptor			0.84
Positive	67.8%	80%	
Negative	32.2%	20%	
Progesterone receptor			0.35
Positive	53%	30%	
Negative	47%	70%	
HER-2 status			0.99
Positive	70.1%	70%	
Negative	29.9%	30%	
Palpable tumour			0.07
Yes	93.6%	70%	
No	6.4%	30%	
Lymph node status			0.04*
Negative	58.2%	80%	
Positive	41.8%	20%	
Tumour type			0.93
Invasive ductal carcinoma (IDC)	72.9%	80%	
Ductal carcinoma in situ (DCIS)	5.7%	0.0%	
Lobular	15.7%	20%	
D & L	1%	0.0%	
Other	2.9%	0.0%	
Histological grade			0.36
I	11.1%	0%	
II	51.9%	70%	
III	37%	30%	
Location			0.04*
Unilateral	95.5%	80%	
Bilateral	2.5%	20%	
Familial history			0.001*
Yes	45%	100%	
No	55%	0%	
Size (cm)			0.64
< 2	59%	60%	
> 2	41%	40%	
Vascular invasion			0.21
Yes	81%	0%	
No	19%	100%	

* p value < 0.05

**Fig. 1:**
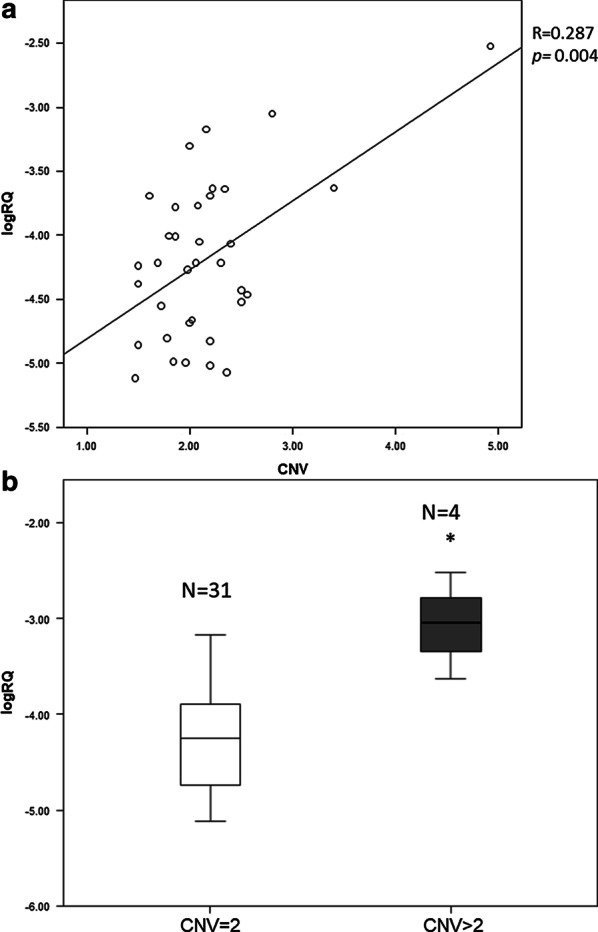
Scatter plots and box plot illustrating respectively the associations between exact CNVs and *E2F1* expression (**a**) and the different *E2F1* expression between patients with CNV = 2 (N = 31) and CNV > 2 (N = 4) in somatic tissue (**b**). *p value < 0.001

Gene expression analysis on breast cancer tissue of 35 out 222 patients (N = 4 with CNV > 2 and N = 31 with CNV = 2 in blood) (Table 3) has revealed a positive and statistically significant correlation between *E2F1* expression and number of copies (*p* = 0.004) (Fig. 1a). Specifically, three out four individuals with germline CNV > 2 showed more copies also in breast tissue.

**Table 3:** Description of phenotypic characteristics of 35 out of 222 patients on which *E2F1* expression and copy number assay were performed

	CNV = 2, (N = 31)	CNV > 2, (N = 4)	*P* value
Age at diagnosis (years)	60.48 ± 16.76	57.5 ± 10.88	0.808
Menopausal status			0.458
Premenopausal	25.8%	0%	
Perimenopausal	3.2%	0%	
Menopausal	71.0%	100%	
Estrogen receptor			0.523
Positive	83.9%	100%	
Negative	16.1%	0%	
Progesterone receptor			0.454
Positive	86.2%	100%	
Negative	19.4%	0%	
HER-2 status			0.082
Positive	74.2%	25%	
Negative	25.8%	75%	
Palpable tumour			0.601
Yes	87.1%	100%	
No	12.9%	0%	
Lymph node status			0.082
Negative	25.8%	75%	
Positive	74.2%	25%	
Tumour type			0.791
Invasive ductal carcinoma (IDC)	80.6%	75%	
Ductal carcinoma in situ (DCIS)	0%	0%	
Lobular	19.4%	25%	
D & L	0%	0%	
Other	0%	0%	
Histological grade			0.304
I	9.7%	0%	
II	35.5%	75%	
III	54.8%	25%	
Location			0.114
Unilateral	100%	75%	
Bilateral	0%	25%	
Familial history			**< 0.001**
Yes	6.5%	100%	
No	93.5%	0%	
Size			0.530
< 2 cm	38.7%	50%	
> 2 cm	61.3%	50%	
Vascular invasion			0.553
Yes	29%	0%	
No	71%	100%

Furthermore, expression levels were significantly different between subjects with CNV = 2 and subjects with CNV > 2 (*p* < 0.001) (Fig. 1b).

## Discussion

BC is the most common malignant tumour among women in the majority of the developed countries and it is normally distinguished into four main molecular subtypes. Although established risk factors are associated with each subtypes (Yang et al. 2007; Sisti et al. 2016; Holm et al. 2017), however, the constant research of novel biomarkers is of fundamental importance in clinical practice so that the patients may have an early diagnosis and specific treatment.

Approximately 5–10% of breast cancers are hereditary and up to 25% are due to mutations in the following high-penetrance genes: *BRCA1, BRCA2, PTEN, TP53, CDH1*, and *STK11* (Oosterwijk et al. 2014; Shiovitz and Korde 2015). Therefore, molecular screening programmes are of huge relevance in terms of prevention, especially in women with BC family history. Indeed, individuals having first degree relatives with genetic mutations, mainly in *BRCA1* and *BRCA2* genes, have an increased risk of developing BC (Ripperger et al. 2009).

Over last years, several groups have studied germinal CNVs as predisposing factors to cancer. CNVs are structural variations ranging from 1 kb to several Mb in length, referred to as polymorphisms if present in more than 1% of population. However, rare CNVs have been described in some tumours, including breast cancer (Kumaran et al. 2017), suggesting a role of these variants as susceptibility factors (Lee and Scherer 2010).

Recently, germinal CNVs of *E2F1* have been proposed as genetic risk factor for testicular cancer and melanoma (Rocca et al. 2017; Rocca et al. 2019). In both studies, patients showed a higher frequency of altered E2F1 copies compared to controls.

E2F1 is a member of E2F family and acts as a transcriptional factor of genes encoding proteins implicated in cell-cycle progression and apoptosis (Attwool et al. 2004). *E2F1* expression has been found to be increased in breast cancer tissue, pointing out the important contribution of this transcriptional factor in breast carcinogenesis (Li et al. 2018).

Based on large body evidence regarding the role of CNV in disease susceptibility and the altered expression of *E2F1* in breast cancer, we have analysed CNVs of *E2F1* in a cohort of 222 women with breast cancer.

Interestingly, we found that the frequency of CNVs of *E2F1* was higher in the patients compared to controls (4.5%), suggesting therefore that altered CNVs of *E2F1* may play a key role as predisposing factor of BC as well as it was reported in patients with testicular cancer and melanoma (Rocca et al. 2017; Rocca et al. 2019). This finding, intriguingly, is corroborated by gene expression analysis that has pointed out a higher expression of *E2F1* in breast tissue from three out of four patients with more germline copies of the gene, included in somatic tissue analysis. Therefore, it is likely that inherited germinal CNVs, in combination with other extrinsic or intrinsic factors, may contribute to increase genomic instability in breast tissue, resulting in the deregulation of E2F1 target proteins.

Patients with more copies of *E2F1* were almost similar to patients with normal copies, except for three clinical characteristics. Indeed, we found a statistically significant difference among two groups for following parameters: lymph nodes status, laterality and family history.

Contralateral breast cancer (CBC) is less common than unilateral breast cancer (UBC) and it has been supposed to have mainly a genetic cause (Mack et al. 2002). Indeed, women carrying mutations in *BRCA1*, *BRCA2* and *CHECK* genes have a higher risk of developing CBC and the estimation of the risk is higher in carriers of germinal mutations, confirming the strong genetic contribution (Robson et al. 2017; Kramer et al. 2020). Based on these evidence, the finding of altered *E2F1* CNVs in patients with CBC suggests that this structural variant, likely inherited, as well as it occurs in inherited germline mutations of BRCA genes, may contribute to an increased risk of CBC. This hypothesis, hence, could explain the early age at diagnosis and BC family history of one of the patients with more copies of *E2F1* and CBC.

It is well known, indeed, that hereditary cancers are generally characterized from an earlier age of onset of BC (Brandt et al. 2008); therefore, the combination of early age of onset and positive BC family history represents a very strong risk factor and is generally associated with germline mutation in BRCA1 gene (Anders et al. 2009).

Interestingly, the patient harbouring more copies of *E2F1* with CBC and positive family history was also carrier of a mutation in BRCA1 gene. This finding could suggest that carriers of inherited rare germline mutations and CNVs of *E2F1* may have an increased risk of BC compared to the individuals having only one of these two genetic variants. It is known that BRCA1 plays a crucial role in the preserving genome integrity, hence, the loss of function of BRCA1 could promote cell cycle progression also of those cells with more copies of *E2F1*, translating into the deregulation of *E2F1* target genes due its overexpression.

In this study, additionally, we have found a significant difference between the two groups of the patients in relation to the presence of lymph nodes positive. Although we have not enough data to clarify this result, we could suppose that this parameter may contribute to better delineate the phenotype linked to the patients having more copies of *E2F1*.

In conclusion, although this study adds information to the research of new biomarkers of BC, the small size of samples and the missing data about the inheritance pattern of the identified CNVs is a limitation, therefore, these results should be confirmed from study on a large cohort of women selected for BC family history.

However, based on our results, we suppose that the high frequency of *E2F1* CNV might predispose to BC, therefore, molecular screening of this gene, mainly in women with positive family history, would be of fundamental importance to estimate the risk of recurrence into the family. Furthermore, the confirmation of germinal CNVs of *E2F1* as novel predictive biomarkers of BC could have impressive implications in clinical practice regarding the choice of targeted therapies against this malignant cancer.

## Conclusions

In conclusion, although this study adds information to the research of new biomarkers of BC, the small size of samples and the missing data about the inheritance pattern of the identified CNVs is a limitation, therefore, these results should be confirmed from study on a large cohort of women selected for BC family history.

However, based on our results, we suppose that the high frequency of *E2F1* CNV might predispose to BC, therefore, molecular screening of this gene, mainly in women with positive family history, would be of fundamental importance to estimate the risk of recurrence into the family. Furthermore, the confirmation of germinal CNVs of *E2F1* as novel predictive biomarkers of BC could have impressive implications in clinical practice regarding the choice of targeted therapies against this malignant cancer.

## Data Availability

All data generated or analysed during this study are included in this published article.

## Abbreviations

CNVs: Copy number variations
BC: Breast cancer
ER: Estrogen receptor
PR: Progesterone receptor
HER2: Human epidermal growth factor
TNBC: Triple negative breast cancer
CBC: Contralateral breast cancer
UBC: Unilateral breast cancer
